# Suppressing Energetic Disorder Enables Efficient Indoor Organic Photovoltaic Cells With a PTV Derivative

**DOI:** 10.3389/fchem.2021.684241

**Published:** 2021-05-12

**Authors:** Pengqing Bi, Junzhen Ren, Shaoqing Zhang, Tao Zhang, Ye Xu, Yong Cui, Jinzhao Qin, Jianhui Hou

**Affiliations:** ^1^School of Chemistry and Biology Engineering, University of Science and Technology Beijing, Beijing, China; ^2^State Key Laboratory of Polymer Physics and Chemistry, Beijing National Laboratory for Molecular, Institute of Chemistry Chinese Academy of Sciences, Beijing, China; ^3^School of Chemical Sciences, University of Chinese Academy of Sciences, Beijing, China

**Keywords:** organic photovoltaics, indoor applications, energetic disorder, PTV, simple chemical structure

## Abstract

Indoor organic photovoltaics (IOPVs) cells have attracted considerable attention in the past few years. Herein, two PTV-derivatives, PTVT-V and PTVT-T, were used as donor materials to fabricate IOPV cells with ITCC as the acceptor. The preferred orientation of the crystals changed from edge-on to face-on after replacing the ethylene in the backbones of PTVT-V by the thiophene in that of PTVT-T. Besides, it was found that, the energetic disorder of the PTVT-T:ITCC based system is 58 meV, which is much lower than that of PTVT-V:ITCC-based system (70 meV). The lower energetic disorder in PTVT-T:ITCC leads to an efficient charge transfer, charge transport, and thus the weak charge recombination. As a result, a PCE of 9.60% under AM 1.5 G and a PCE of 24.27% under 1,000 lux (LED 2,700 K) with a low non-radiative energy loss of 0.210 eV were obtained based on PTVT-T:ITCC blend. The results indicate that to improve the PTV-derivatives photovoltaic properties by suppressing the energetic disorder is a promising way to realize low-cost IOPV cells.

## Introduction

The advantages of light weight, flexibility, and roll-to-roll large scale production method enable organic photovoltaic (OPV) technique becomes one of the promising clean energy candidates in the future (Yu et al., 1995; Kaltenbrunner et al., 2012; Li et al., 2018). The OPV cells have experienced rapid development during the past few decades due to the multi-disciplinary efforts, including the design of new materials, the device physics, and the device engineering (Clarke and Durrant, 2010; Meng et al., 2018; Menke et al., 2018; Qian et al., 2018; Yan et al., 2018; Yuan et al., 2019; Cui et al., 2020b), as a result, the record power conversion efficiency (PCE) of single-junction OPV cell has exceeded 18% (Liu et al., 2020a; Wang, 2020; Zhang et al., 2021). Besides the outdoor applications, OPV cells under dim light or indoor light conditions also show great potential in the application of off-grid energy sources (Cui et al., 2019; Singh et al., 2020; Zhang et al., 2020). Indoor OPV (IOPV) cells can convert the energy emitted from the household lighting facilities or ambient dim-light sources into the energy at the scale of megawatt to microwatt, showing great potential in supplying the power to wireless electronics such as smart housing sensors and sensors for the “Internet of Things.”

To rationally design photoactive materials for the application of IOPV cells, first of all, the photoresponse spectrum of the light-harvesting materials should match that of the indoor light sources. Since the illumination spectra of fluorescence lamps and light-emitting diodes (LEDs) is mainly in range from 400 to 700 nm, in comparison of inorganic photoactive materials such as silicon, the advantages of highly tunable absorption spectra and relatively high coefficient of OPV materials enable IOPV becomes a promising candidate for the application of indoor energy. The IOPV cells have received considerable attention in recent years and the PCEs were improved rapidly (Cui et al., 2020a; Ma et al., 2020b). However, it still faces some critical challenges in molecular design of photovoltaic materials. For instance, the charge carrier densities of the OPV cells under room light sources are significantly lower than that of the cells under 1 sun irradiation due to the low illumination intensities. As recombination centers, trap-state plays a key role in determining the performance of IOPV cell. For the application of OPV cells under the illumination of one sun, the trap-state density, *ca*. 10^−15^ cm^−3^, is negligible compared to the density of photogenerated charge carriers (Ma et al., 2020a). In contrast, for the device under dim light sources, the density of photogenerated charge carriers is relatively low, which is even comparable to that of the trap-state density (Cui et al., 2020a). A large proportion of photogenerated charge carriers are caught in the “traps” and do not attributed to photocurrent generation. Therefore, the performance of OPV cells under dim light sources is very sensitive to the trap-state.

For the OPV materials, the trap-states can be mainly divided into two categories, i.e., intrinsic and extrinsic traps (Kotadiya et al., 2018, 2019; Haneef et al., 2020). Considerable research efforts have been devoted to control the extrinsic traps, resulting in the obvious performance enhancement in the corresponding systems (Zuo et al., 2017; Song et al., 2018; He et al., 2019; Yan et al., 2019). The intrinsic traps are directly related to the energetic disorder of a certain material system. Compared to the inorganic counterparts, organic molecules demonstrate relatively high energetic disorder due to the presence of structural inhomogeneities (Liu et al., 2020b). Thus, it is of great importance to explore the relationship among the chemical structures of photoactive materials, the trap-state density, and the photovoltaic properties under indoor light applications. Besides of the improvement of PCEs, cost is also a significant consideration in estimating the application of OPV devices (Xue et al., 2018; Al-Ahmad et al., 2020). In the current period, the cost for fabricating efficient large scale OPV cells is still contrary to the principle of low-cost of OPV technique, because the chemical structures of the efficient materials are relatively complex. In our previous reports, we found that the PTV-derivatives, which have simple structure molecules and exhibit low-cost feature, can work well with the widely used non-fullerene-based acceptors (Bi et al., 2020; Ren et al., 2021), and they may become potential candidates in the application of IOPV.

In this work, IOPV cells with two PTV-derivatives, named PTVT-V and PTVT-T, as the donors, and a wide bandgap acceptor, ITCC, as the acceptor were fabricated, respectively. For the two donors, PTVT-V prefers edge-on orientation and PTVT-T shows face-on dominated orientation. More importantly, the PTVT-T based devices demonstrate much lower energetic disorder than that of PTVT-V. The lower energetic disorder significantly decreased the charge recombination. As a result, a PCE of 24.27%, under the light source of LED 2,700 K, 1,000 lux, was obtained based on the blend of PTVT-T:ITCC, which is much higher than the device based on PTVT-V:ITCC (5.10%). The results indicate that suppressing the energetic order via optimizing the molecular structure is an effective way to achieve high performance IOPV cells.

## Results and Discussion

**Figure 1:**
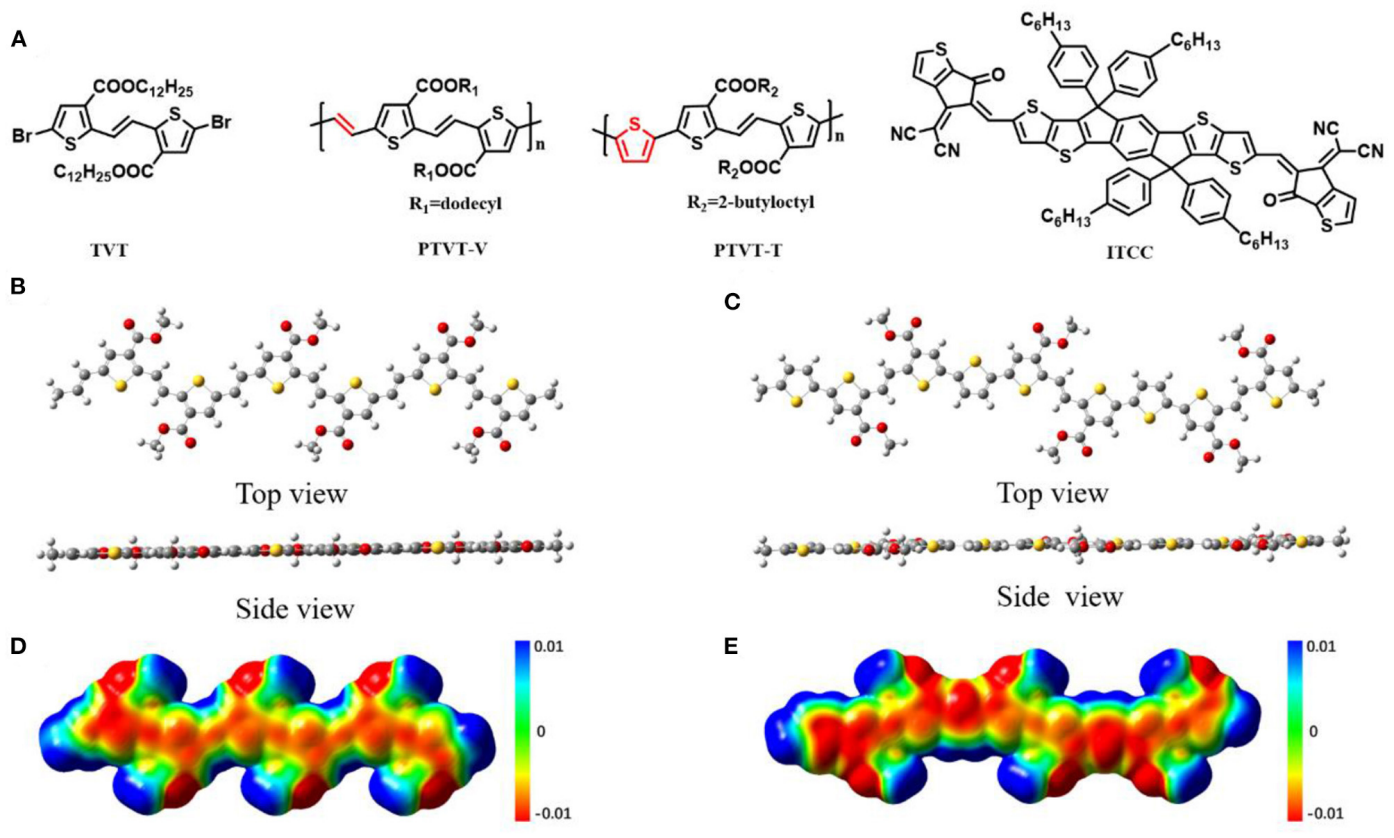
**(A)** Molecular structures of the TVT monomer, PTVT-V, and PTVT-T. The top-view and side-view of optimized geometries of **(B)** PTVT-V and **(C)** PTVT-T. ESP distributions of **(D)** PTVT-V and **(E)** PTVT-T.

The chemical structures of TVT monomer, PTVT-V, PTVT-T, and ITCC are shown in Figure 1A. Density functional theory (DFT) with a level of B3LYP/6-31G (d, p) was used to calculate the molecular properties, including the highest occupied molecular orbital (HOMO) and the lowest unoccupied molecular orbital (LUMO) levels, the molecular geometries, and the surface electrostatic potential (ESP) distribution of both PTVT-V and PTVT-T. The calculated HOMO/LUMO levels of PTVT-V and PTVT-T are −4.97/−2.96 and −4.94/−2.87 eV, respectively, all of which are delocalized over the molecular backbones, as shown in Supplementary Figure 1. Besides, the optimized molecular geometries of both PTVT-V and PTVT-T are shown in Figures 1B,C. The optimized molecular structures of both PTVT-V and PTVT-T demonstrate planar configuration, which are favor to the formation of the ordered molecular packing and thus the efficient charge transport. The ESP distributions of the two molecular models are mapped in Figures 1D,E. PTVT-T shows more negative ESP values on the molecular backbone, which indicates that the PTVT-T has stronger electron-donating feature than PTVT-V (Yao et al., 2019).

**Figure 2:**
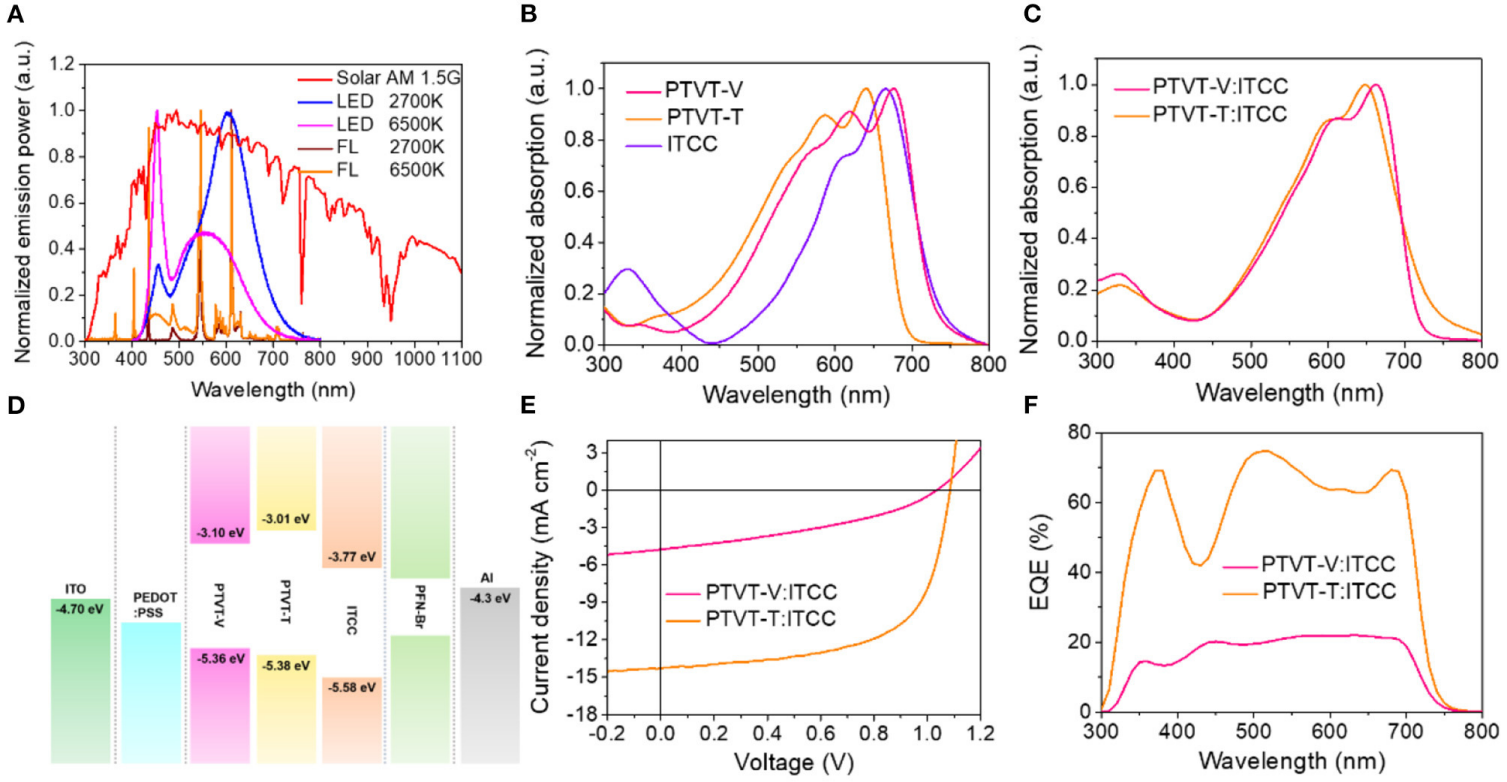
**(A)** The spectra of AM 1.5 G and various indoor light sources. **(B)** The absorption spectra of neat films of PTVT-V, PTVT-V, and ITCC. **(C)** The absorption spectra of blend films of PTVT-V:ITCC and PTVT-T:ITCC. **(D)** Energy level diagrams of the materials used in this work. **(E)** *J-V* curves of the OPV devices based on PTVT-V:ITCC and PTVT-T:ITCC under the illumination of AM 1.5 G, 100 mW cm^−2^. **(F)** The corresponding EQE spectra of the devices.

The differences between OPV cells under sun light and indoor light sources are mainly due to the difference of the radiation spectra, as shown in Figure 2A. Unlike the spectrum of AM 1.5 G, the spectra of the indoor light sources are mainly in the range of 400–700 nm. As mentioned before, the photoactive materials should have matched absorption spectra with the indoor light sources to obtain high performance. The absorption spectra of neat PTVT-V, PTVT-T and ITCC as well as the corresponding blend films are shown in Figures 2B,C. It can be clearly observed that the donors/acceptor materials show absorption spectrum covering the visible range of 450–700 nm, which could meet the requirements of the absorption capability for IOPV application. Compared to the absorption spectrum of PTVT-V, PTVT-T exhibits a *ca*. 50 nm blue shifted absorption spectrum, which is more suitable to fabricate an IOPV cell when blending with ITCC. The cyclic voltammograms (CV) curves and the corresponding calculated energy levels are shown in Supplementary Figure 2 and Figure 2D. The donors and the acceptor show well matched energy levels and the HOMO levels of PTVT-V and PTVT-T are on the same level, which is −5.36 and −5.38 eV, respectively.

**Figure 3:**
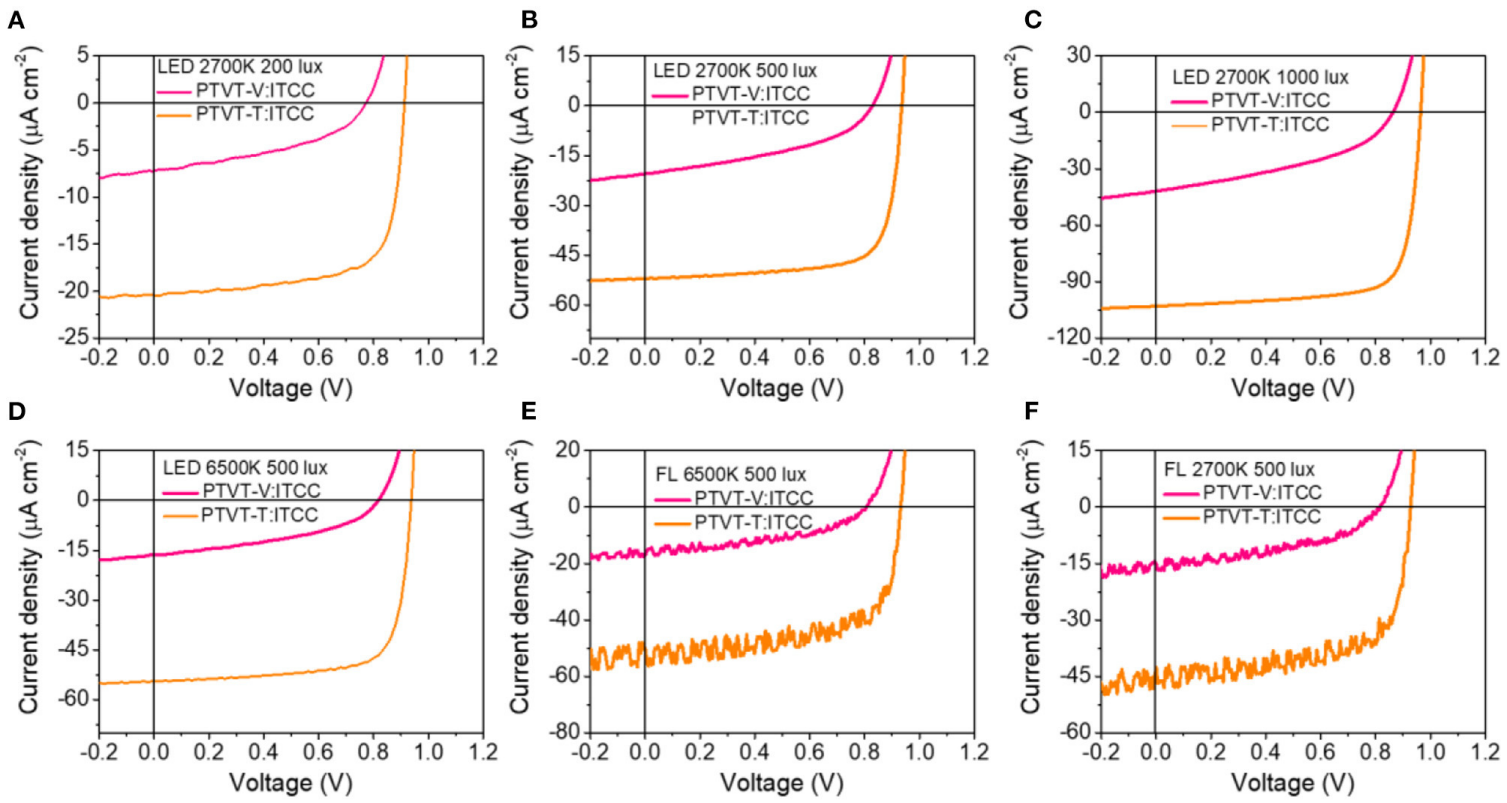
*J-V* curves of the devices based on PTVT-V:ITCC and PTVT-T:ITCC under various light sources with different light intensities of **(A)** LED 2,700 K, 200 lux. **(B)** LED 2,700 K, 500 lux. **(C)** LED 2,700 K, 1,000 lux. **(D)** LED 6,500 K, 500 lux. **(E)** FL 6,500 K, 500 lux. **(F)** FL 2,700 K, 500 lux.

OPV cells based on PTVT-V:ITCC and PTVT-T:ITCC were fabricated in parallel. The current density vs. voltage *(J-V)* curves under the illumination of AM 1.5 G and the corresponding device parameters are shown in Figure 2E and Table 1. The OPV cell based on PTVT-V:ITCC shows a poor PCE of 1.81% with an open-circuit voltage (*V*_OC_) of 1.04 V, a short-circuit current density (*J*_SC_) of 4.76 mA cm^−2^ and a FF of 36.85%. However, the device based on PTVT-T:ITCC demonstrates a much higher PCE of 9.60% with a *V*_OC_ of 1.08 V, a *J*_SC_ of 14.30 mA cm^−2^ and an FF of 62.06%. The external quantum efficiency (EQE) profiles are shown in Figure 2F. The OPV cell based on PTVT-T:ITCC possesses high EQE values in the range of 350–700 nm, which is much higher than the device based on PTVT-V:ITCC. More importantly, the corresponding integrated *J*_SC_ of both OPV cells from the EQE spectra are consistent with the *J*_SC_ that obtained from the *J-V* measurements. The performance of the OPV cells under the indoor light sources were then measured to investigate the effect of the variation in the molecular structures on their device performance. The *J-V* curves of cells under four light sources, LED 2,700 K, LED 6,500 K, FL 2,700 K, and FL 6,500 K, are shown in Figure 3. The corresponding device parameters were summarized in Table 2 and Supplementary Table 1. The intensities of the indoor light sources were measured and calculated following the similar procedure with the previous studies (Cui et al., 2019). Under LED 2,700 K, obvious enhancement in *J*_SC_ and *V*_OC_ can be obtained with the increase of the light intensity from 200 to 1,000 lux, which may be attributed to the increased ratios of photogenerated charge carrier density to trap-state density. For the device based on PTVT-V:ITCC under LED 2700 K 1,000 lux, it demonstrates a highest maximum power density (MPD) of 16.07 μW cm^−2^, with a *V*_OC_ of 0.859 V, a *J*_SC_ of 41.08 μA cm^−2^ and FF of 45.54%, which corresponds to a PCE of 5.10%. While, the OPV cells based on PTVT-T:ITCC shows much higher device performance than that based on PTVT-V:ITCC. Under LED 2,700 K 1,000 lux, the cell based on PTVT-T:ITCC exhibits a MPD of 76.72 μW cm^−2^ and a PCE of 24.27%, with a *V*_OC_ of 0.967 V, a *J*_SC_ of 103.06 μA cm^−2^ and FF of 76.72%. The device performance under LED 6,500 K, FL 2,700 K, FL 6,500 K with intensity of 500 lux were also measured. All of the devices show similar PCEs, which suggests the device can work very well under various illuminations. It should be noted that, the *J*_SC_ values that integrated from the EQE under the indoor light sources are slight difference with the *J*_SC_ values obtained via the *J-V* measurements, which is within the estimated error for the indoor *J-V* measurements, the results are shown in Supplementary Figure 3.

**Table 1:** Photovoltaic parameters of the OPV cells based on PTVT-V:ITCC and PTVT-T:ITCC under the illumination of AM 1.5 G, 100 mW cm^−2^.

**Active layer**	***V*_**OC**_ (V)**	***J*_**SC**_ (mA cm^**−2**^)**	**Cal. *J*_**SC**_ (mA cm^**−2**^)**	**FF (%)**	**PCE (%)^a^**
PTVT-T:ITCC	1.08	14.30	14.00	62.06	9.60 (9.46 ± 0.06)
PTVT-V:ITCC	1.04	4.76	4.43	36.85	1.81 (1.74 ± 0.03)

a *Average values with standard deviation were obtained from 10 devices*.

**Table 2:** Photovoltaic parameters of the devices based on PTVT-V:ITCC and PTVT-T:ITCC under various light sources with different light intensities.

**Light sources**	**Active layer**	**Intensity(lux)**	**P_**in**_ (μW cm^**−2**^)**	***V*_**OC**_(V)**	***J*_**SC**_ (μA cm^**−2**^)**	**Cal. *J*_**SC**_(μA cm^**−2**^)**	**FF (%)**	**P_**out**_(μW cm^**−2**^)**	**PCE^**a**^ (%)**
2,700 K LED	PTVT-V:ITCC	200	60	0.762	7.69	7.28	44.37	2.60	4.3 (4.24 ± 0.05)
		500	156	0.830	19.87	18.62	44.93	7.41	4.75 (4.67 ± 0.08)
		1,000	315	0.859	41.08	39.86	45.54	16.07	5.10 (4.94 ± 0.09)
	PTVT-T:ITCC	200	60	0.910	20.46	19.56	72.23	13.45	22.42 (21.87 ± 0.29)
		500	156	0.936	52.16	50.01	74.40	36.26	23.24 (22.84 ± 0.33)
		1,000	315	0.967	103.06	98.26	76.72	76.46	24.27 (23.89 ± 0.37)

*Average values with standard deviation were obtained from 10 devices*.

Atomic force microscope (AFM) was used to investigate the surface morphology of the neat and blend films. The corresponding height and phase images are shown in Supplementary Figure 4. Obvious granular substance can be seen in the neat PTVT-V film, resulting a relatively high root mean square (RMS) roughness of 6.49 nm. For the neat film of PTVT-T, the large granular substances are absent. It exhibits smooth and ordered aggregation characteristics, which can be attributed to the relatively strong aggregation effect of PTVT-T (Ren et al., 2021). Similar trend was observed in the blend films. The granular substances of PTVT-V still existed in the PTVT-V:ITCC blend, and there is no phase separation effect can be observed in the blend film, which may be caused by the excessive miscibility between the PTVT-V and ITCC. For the blend film of PTVT-T:ITCC, obvious phase separation can be observed.

**Figure 4:**
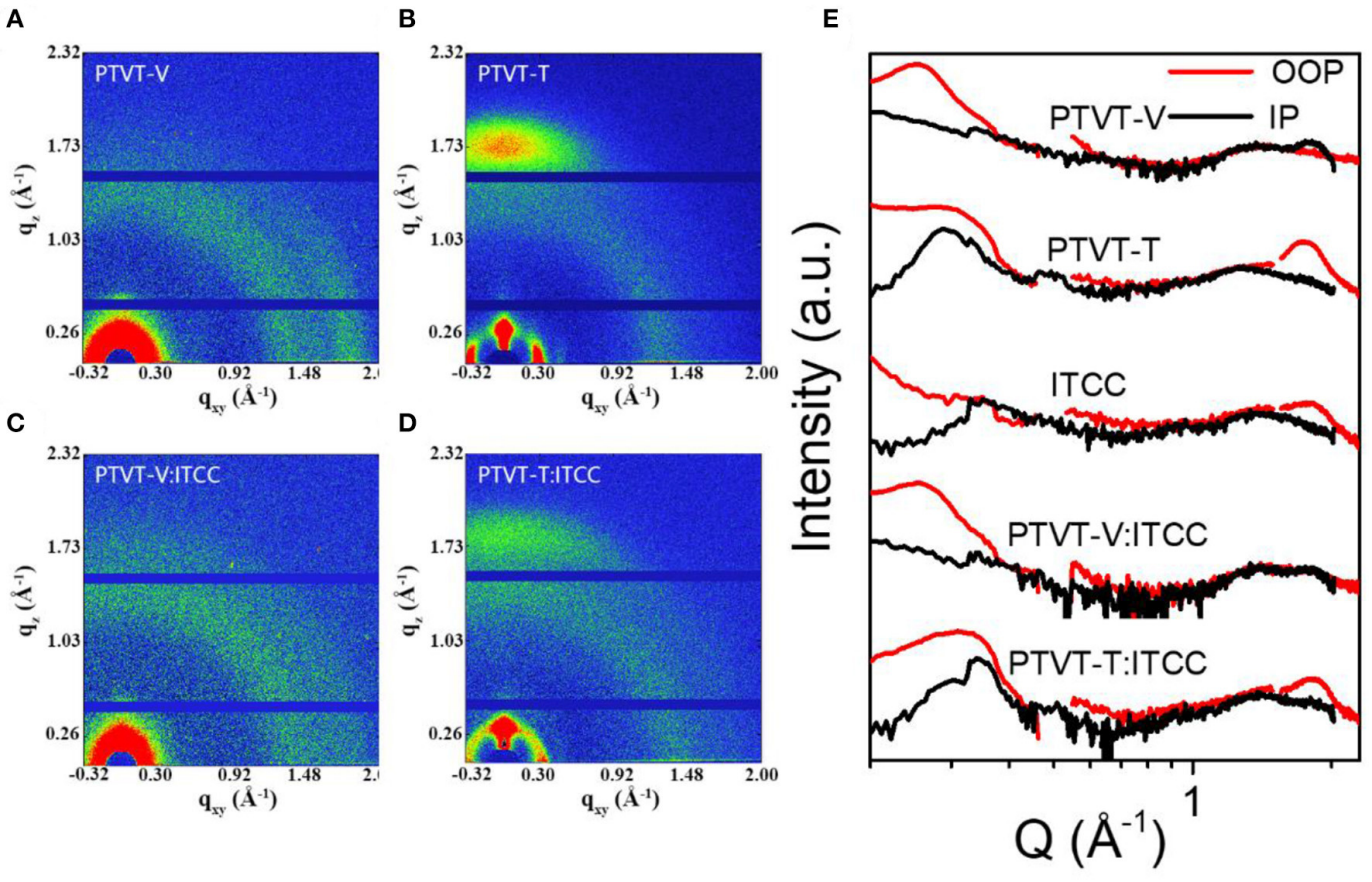
The 2D scattering patterns of **(A)** PTVT-V, **(B)** PTVT-T, **(C)** PTVT-V:ITCC and **(D)** PTVT-T:ITCC. **(E)** The corresponding OOP and IP GIWAXS profiles of both neat and blend films.

Grazing incidence wide-angle X-ray scattering (GIWAXS) was performed to further investigate the packing properties of the photoactive molecules. The 2D GIWAXS patterns and corresponding 1D profiles obtained along in-plane (IP) and out-of-plane (OOP) directions are shown in Figure 4 and Supplementary Figure 5a. For the neat film of PTVT-T, it demonstrates a strong (010) π-π stacking peak in the OOP direction without any signals in the IP direction, which means PTVT-T has a face-on dominated orientation (Bi et al., 2018). For the pristine PTVT-V, compared to PTVT-T, weaker (010) π-π stacking peak can be found in the IP direction and no obvious (010) peak in the OOP direction, indicating that PTVT-V prefers an edge-on orientation. The face-on molecular orientation in PTVT-T is more favorable for the charge transport in the vertical structure devices compared to the edge-on orientation in PTVT-V. However, the blend film based on PTVT-V:ITCC shows no obvious crystal orientation. In the blend films, it can be seen that the face-on molecular orientation of PTVT-T can be maintained in the PTVT-T:ITCC blend. Furthermore, the locations of the π-π stacking peaks of PTVT-V:ITCC and PTVT-T:ITCC are 1.79 and 1.84 Å^−1^, corresponding to the *d*-spacing of 3.53 and 3.41 Å, respectively. The stronger molecular packing property and smaller *d*-spacing in the PTVT-T:ITCC blend film than that of PTVT-V:ITCC blend film is more favorable for efficient charge transport.

**Figure 5:**
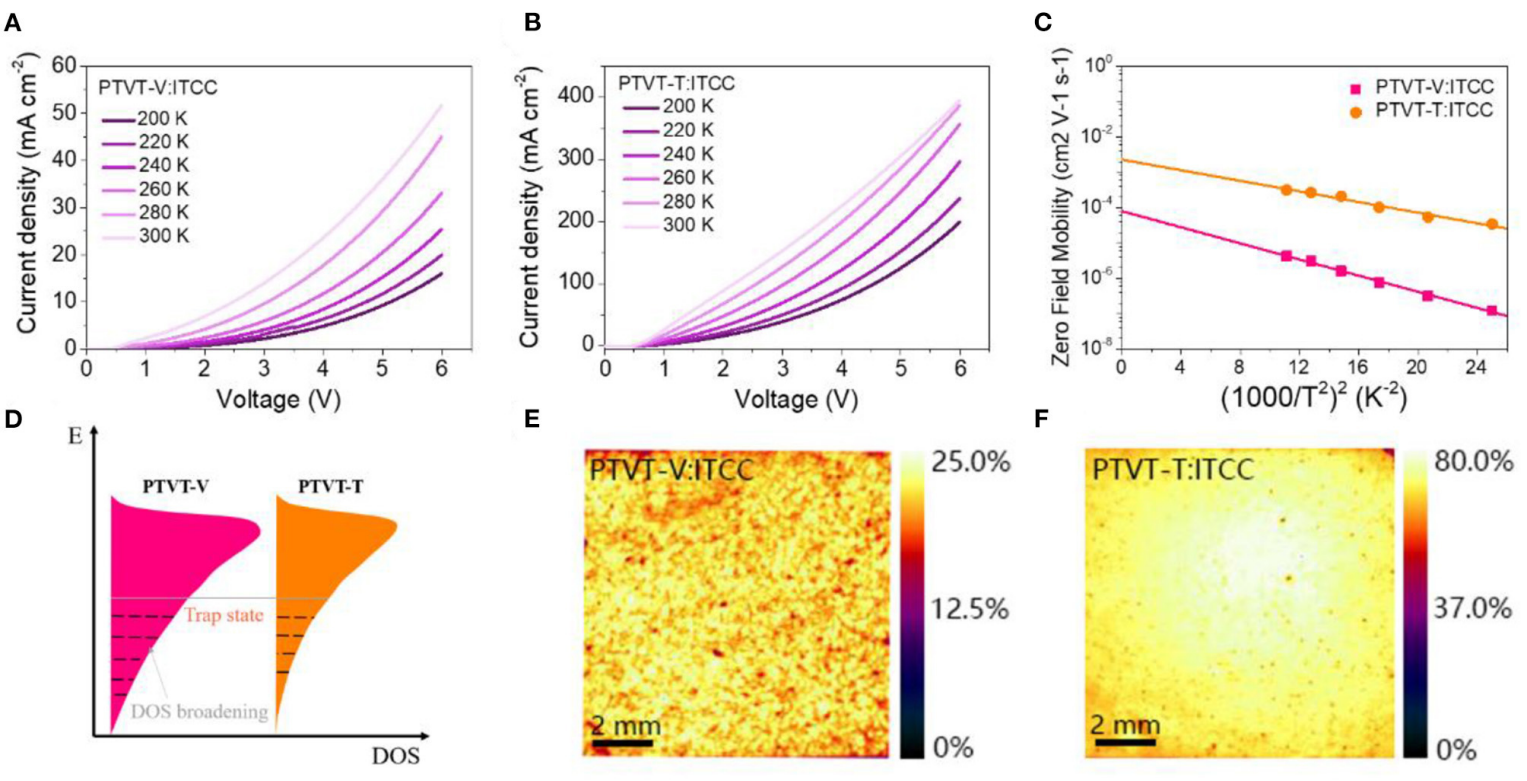
SCLC plots under different temperatures for the devices based on **(A)** PTVT-V:ITCC and **(B)** PTVT-T:ITCC. **(C)** The plots of hole mobilities of the devices as a function of 1/T^2^ using SCLC derived data. **(D)** The diagram of the density of the state (DOS) of HOMO levels of PTVT-V and PTVT-T. The EQE mapping of the blend devices of **(E)** PTVT-V:ITCC and **(F)** PTVT-T:ITCC.

In order to investigate the origin of the differences in device performance, space-charge limited current (SCLC) method was used to measure the hole mobilities of these two systems at various temperatures. The hole mobilities of these two systems can be obtained via fitting the *J-V* curves as shown in Figures 5A,B. At room temperature (*T* = 300 K), the hole mobility of the OPV cell based on PTVT-T:ITCC is 2.61 × 10^−4^ cm^−2^ v^−1^ s^−1^, which is much higher than that of PTVT-V:ITCC (3.00 × 10^−6^ cm^−2^ v^−1^ s^−1^). It can be reasonable speculated that the higher FF of PTVT-T:ITCC-based device than that of PTVT-V:ITCC-based one can ascribe to the higher hole mobilities of PTVT-T:ITCC. Gaussian disorder model (GDM) was further used to reveal the electronic states of hole transport in the both PTVT-V:ITCC and PTVT-T:ITCC systems. The relation between zero-field hole mobility and temperature can be expressed as follows:

$$\begin{array}{c} \mu_{0}=\mu_{\infty }\exp\left[-{\left(\frac{2\sigma }{3kT}\right)}^{2}\right]\; \end{array}$$

Where μ_∞_ is the hole mobility at infinite temperature, *k* is the Boltzmann constant, and σ is the energetic disorder. The plots of zero-field mobilities of holes as a function of $\frac{1}{T^{2}}$ are shown in the Figure 5C. It was shown that, σ can be used to describe the energetic spreading of the HOMO sites, i.e., the energetic disorder (Liu et al., 2020b; Lv et al., 2020). The smaller value of σ means fewer trap states near the HOMO levels. The calculated values of σ of PTVT-V:ITCC and PTVT-T:ITCC systems are 70 and 58 meV, respectively, as shown in Supplementary Table 2. The results show that more trap states existed in the tailed states of PTVT-V:ITCC than that of PTVT-T:ITCC system, as shown in diagram of Figure 5D. The free charge carriers can be captured by the trap sates and recombined with the electrons, which is a huge energy loss for the real working OPV cells under dim light sources. In order to clearly show the effect of trap states on charge generation and transport, we further measured the EQE distribution (1 cm^2^) with an excitation light of 520 nm laser. As displayed in Figures 5E,F. The EQE distribution for the device based on PTVT-T:ITCC is much more uniform than that of PTVT-V:ITCC, which suggests that PTVT-T:ITCC-based device has higher efficient charge generation and collection than the PTVT-V:ITCC-based device.

**Figure 6:**
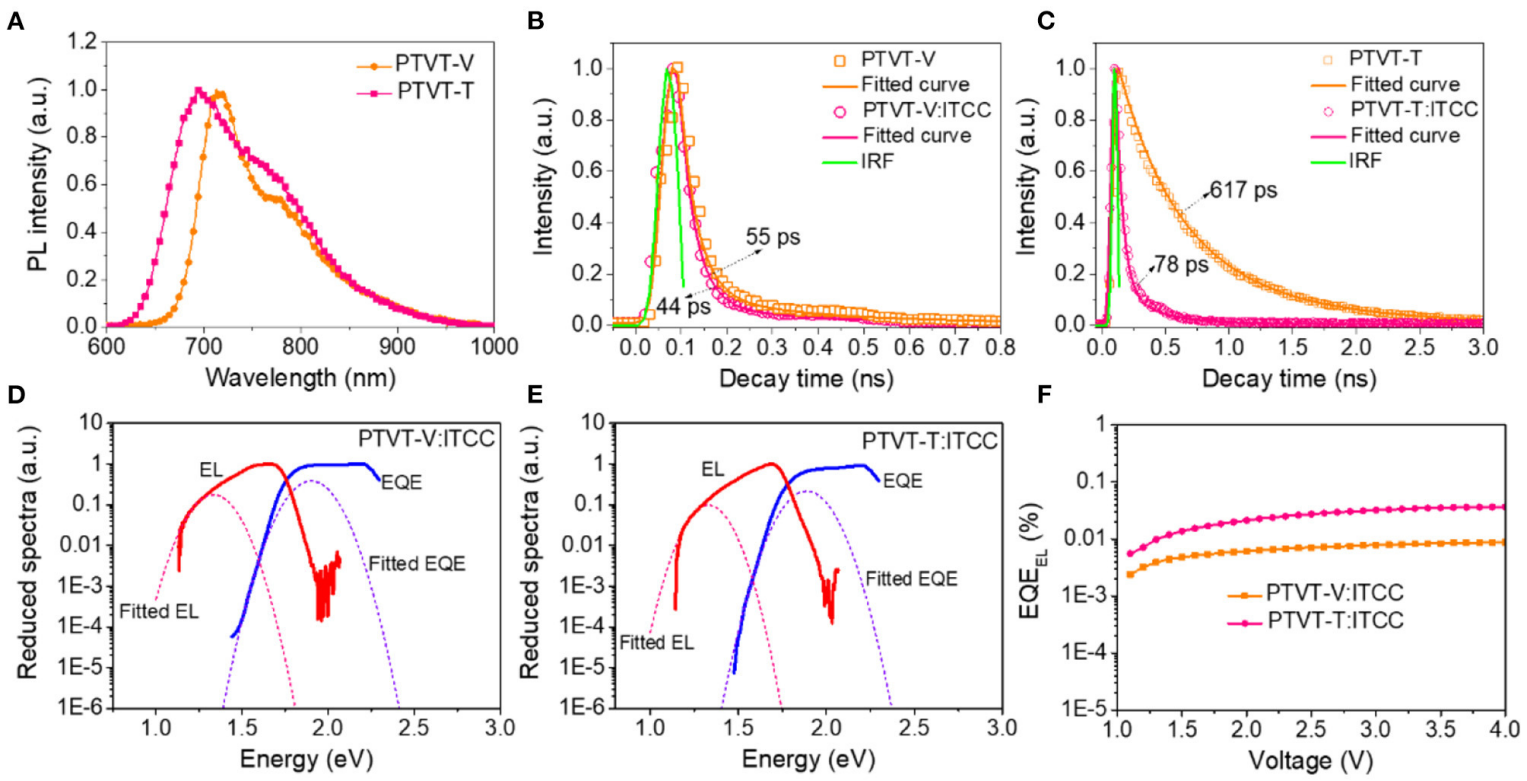
**(A)** The steady-state PL spectra of PTVT-V and PTVT-T neat films with an excitation wavelength of 500 nm. The PL decay kinetics of **(B)** PTVT-V based systems and **(C)** PTVT-T based systems. The determination of *E*_CTs_ by EL and s-EQE spectra of **(D)** PTVT-V:ITCC and **(E)** PTVT-T:ITCC. **(F)** *EQE*_EL_ of the devices based on PTVT-V:ITCC and PTVT-T:ITCC.

The charge transfer dynamics in both systems were further characterized by time resolved photoluminescence (TRPL). According to the absorption spectra, an excitation light at 500 nm was used to mainly excite the donors of PTVT-V and PTVT-T. The PL and TRPL spectra are shown in Figure 6. The fluorescence lifetime of PTVT-V and PTVT-T neat films are 55 and 617 ps, respectively. The longer fluorescence lifetime of PTVT-T indicates the relative weak charge recombination processes in the system of PTVT-T:ITCC. After blending with ITCC, the fluorescence lifetimes of PTVT-V:ITCC and PTVT-T:ITCC decay to 44 and 78 ps, respectively. The faster decay time means that the charge transfer between donor and acceptor in PTVT-T:ITCC is more efficient than the system of PTVT-V:ITCC, resulting in smaller charge recombination in the system of PTVT-T:ITCC (Bi et al., 2019).

As discussed before, the energetic disorder plays a key role in determining the intrinsic traps, as well as the *V*_OC_ loss, of photovoltaic materials with certain chemical structures. Compared to the OPV cells using the incident light of one sun, the *V*_OC_ loss is much more important to the OPV cells working under dim light. Here, the *V*_OC_ loss was measured carefully by using highly sensitive external quantum efficiency (HEQE) spectrum and electroluminescence (EL). The sEQE and EL spectra of these two systems are shown in Figures 6D,E. The detailed energy loss parameters are shown in Table 3. The blend systems of PTVT-V:ITCC and PTVT-T:ITCC have the same calculated optical bandgap of 1.78 eV, as shown in Supplementary Figure 5. The radiative energy loss of PTVT-V:ITCC and PTVT-T:ITCC are 0.208 and 0.204 eV, respectively. However, the non-radiative, which is closely related to the charge recombination, can be quantitively calculated via the results of EQE_EL_ (Hou et al., 2018). As shown in Figure 6F, the EQE_EL_ of these two devices are ca. 3 × 10^−4^ and 7 × 10^−5^, corresponding to a *E*_3_ of 0.246 and 0.210 eV for PTVT-V:ITCC- and PTVT-T:ITCC-based device, respectively. The relative larger non-radiative energy loss in PTVT-V:ITCC-based cell should be resulted from the increased energetic disorder that causes the free charge carriers to be easily trapped by the trap states.

**Table 3:** Detailed energy loss parameters of the OSCs based on PTVT-V and PTVT-T.

**Blends**	**ΔE_*g*_(*eV*)**	**E (eV)**	${\text{V}}_{OG}^{SQ}$ **(V)**	**ΔE_1_ (eV)**	**ΔE_2_ (eV)**	**ΔE_3_ (eV)**
PTVT-V:ITCC	1.78	0.74	1.494	0.286	0.208	0.246
PTVT-T:ITCC	1.78	0.70	1.494	0.286	0.204	0.210

Unlike the high-performance photovoltaic materials, the PTV derivatives possess very simple molecular structures without fused ring and expensive substituent functional groups. As reported in our previous work, PTVT-T can be easily obtained in five steps. The plot of PCEs of IOPV cells as a function of the steps that required for material synthesis are shown in Supplementary Figure 6a. The chemical structures and detailed parameters of the IOPV cells are shown in Supplementary Figure 6c and Supplementary Table 3. It can be easily found that PTVT-T has the highest PCE for the IOPV cell application with the minimum synthesis steps. Besides, the IOPV device with a relatively large area (4 cm^2^) was fabricated, the cell shows a PCE of 22.34%, with a *V*_OC_ of 0.927 V, a *J*_SC_ 50.87 μA cm^−2^ of and a FF of 73.93%. The photograph of the device and its *J-V* characteristic are shown in Supplementary Figure 6b. Despite the relatively large device area, the device still exhibits a PCE of 24% under the light source of LED 2,700 K with an intensity of 500 lux.

## Conclusion

PTVT-V and PTVT-T are synthesized based on TVT monomer and both show very simple chemical structures. PTVT-V prefers an edge-on orientation, whereas PTVT-T prefers face-on orientation, which is more favorable for the charge transport. More importantly, PTVT-T was found has much lower energetic disorder (58 meV) in compression to that of PTVT-V (70 meV). The smaller energetic disorder can enable the blend of PTVT-T:ITCC has low trap-state density, which can effectively suppress the charge recombination processes. For the real devices, the small energetic disorder leads a relatively small non-radiative energy loss in the OPV cell based on PTVT-T:TCC. Finally, the OPV cells based on PTVT-V:ITCC and PTVT-T:ITCC demonstrate the MPDs (PCEs) of 7.41 μW cm^−2^ (4.75%) and 36.26 μW cm^−2^ (23.24%) under the illumination of LED 2,700 K, 500 lux, respectively. A PCE of 24.27% can be realized in PTVT-T:ITCC-based IOPV cell under 1,000 lux (LED 2,700 K). This work demonstrates that conjugated polymers with simple molecular structures and low energetic disorder is with great importance for the future indoor light applications of OPV cells.

## Data Availability Statement

The original contributions generated for the study are included in the article/Supplementary Material, further inquiries can be directed to the corresponding author.

## Author Contributions

PB, SZ, and JH conceived the idea. PB carried out the materials selection, cells fabrication, and characterizations. JR synthesized the donor materials. The IOPV performance was measured with the help of YX and YC. TZ and JQ conducted the AFM characterizations. All authors discussed and commented on the key scientific issues in the work.

## Conflict of Interest

The authors declare that the research was conducted in the absence of any commercial or financial relationships that could be construed as a potential conflict of interest. The reviewer ZK declared a shared affiliation, with no collaboration, with the authors PB, JR, TZ, YX, YC, JQ, JH to the handling editor at the time of the review.
