# Supplementary material for: Suppressing Energetic Disorder Enables Efficient Indoor Organic Photovoltaic Cells With a PTV Derivative
Source: Front Chem. 2021 May 12;9:684241. doi: 10.3389/fchem.2021.684241 (PMC8149913; doi:10.3389/fchem.2021.684241)
Supplement: Supplementary file 1 [file Data_Sheet_1.PDF]

Supporting information

Suppressing Energetic Disorder Enables Efficient Indoor Organic Photovoltaic cells with a PTV Derivative

Pengqing Bi,[1, 2] Junzhen Ren,[1, 2] Shaoqing Zhang,[1*] Tao Zhang,[2] Ye Xu,[2, 3] Yong Cui,[2] Jinzhao Qin,[2, 3] Jianhui Hou[1, 2, 3]

[1]School of Chemistry and Biology Engineering, University of Science and Technology Beijing, Beijing 100083, P. R. China

[2]State Key Laboratory of Polymer Physics and Chemistry, Beijing National Laboratory for Molecular, Institute of Chemistry Chinese Academy of Sciences, Beijing 100190, P. R. China

[3]University of Chinese Academy of Sciences, Beijing 100049 (P. R. China)

*E-mail: shaoqingz@iccas.ac.cn (S. Z.)

1. Experimental Section

1.1 Materials

PTVT-V and PTVT-T were synthesized according to our previous reports.(Bi et al., 2020) The molecular weight (Mn) of PTVT-V and PTVT-T are 11.2K and 37.0K with polydispersity (PDI) of 2.2 and 2.3, respectively. ITCC and PFN-Br were purchased from Solarmer Materials, Inc. and used as received without further purification.

1.2 Characterization of materials

Hitachi UH5300 spectrophotometer was used to collect the absorption spectra. CV measurements were used to measure the energy levels through a CHI650D electrochemical workstation. AFM images were measured via a Bruker Nanoscope V AF microscope in tapping mode. PL spectra were collected by a spectrometer (DU420A-OE, ANDOR) with an excitation wavelength of 500 nm. TRPL spectra were measured through TCSPC technique (Becker & Hickl, SPC-150) with an excitation wavelength of 500 nm. The instrument response function (IRF) of the setup is *ca.* 400 fs. The hole mobilities were measured by SCLC method with a hole-only device structure of ITO/PEDOT:PSS/Active layers/Au. Mott-Gurney law was used to fitted the J-V curves of the hole-only devices, which was described as follows:

$$J = 9\varepsilon_0\varepsilon_r\mu V^2/8d^3$$

Where, J is the current density, ε_r is the dielectric permittivity of the active layer, ε_0 is the vacuum permittivity, d is the thickness of the active layer, and μ is the mobility. V is the effective voltage, which can be obtained through $V = V_{app} - V_{bi}$, where V_{app} is the applied voltage, and V_{bi} is the offset voltage. The hole mobilities can be calculated from the slope of the $\ln(JL^3V^2) - V^{0.5}d^{0.5}$ curves.

GIWAXS were measured on a Xeuss 2.0 SAXS/WAXS system (Xenocs SA, France). The wavelength of the X-ray radiation is 1.5418 Å. A semiconductor detector (Pilatus 300 K, DECTRIS, Swiss) was used to collect the scattering signals. The incident angle was 0.2°. s-EQE was measured using an integrated system (PECT-600, Enlitech) EQE_{EL} and EL spectra were collected by applying external voltage (0-4 V) through the devices (ELCT-3010, Enlitech). The devices used for external quantum efficiency of electroluminescence (EQE_{EL}) and EL spectra measurements were fabricated according to the optimized conditions.

1.3 Devices fabrication and characterization

A conventional structure of ITO/PEDOT:PSS/Active layers/PFN-Br/Al was used to fabricate the OPV cells. ITO-coated glasses were cleaned by ultrasonic treatment in detergent, deionized water, acetone, and isopropanol, respectively. The cleaned ITO substrates were then treated with ultraviolet-ozone for 15 min. PEDOT:PSS was spin coated on the ITO substrates with a thickness of 30 nm. The PEDOT:PSS-coated substrates were then annealed at 150°C for 15 mins under atmosphere. 100 nm thick Active layers with thickness of *ca.* 150 nm were obtained by spin-coating the blend solutions with solvent additives on the PEDOT:PSS layers. The blend films were annealed at 100°C for 10 min. 5 nm PFN-Br from alcohol solution were deposited on the active layers at 3000 rpm. Finally, about 100-nm-thick Al was deposited onto the active layer under vacuum at a pressure below 3×10^{-4} Pa. The effective area of the device is *ca.* 1 cm^2. *J-V* curves characteristics of the OSCs were measured under AM1.5G illumination of 100 mW cm^{-2} (SS-F5-3A, Enlitech) in glove box. The radiative intensity was calibrated by the standard silicon solar cell. EQE was characterized in air condition.

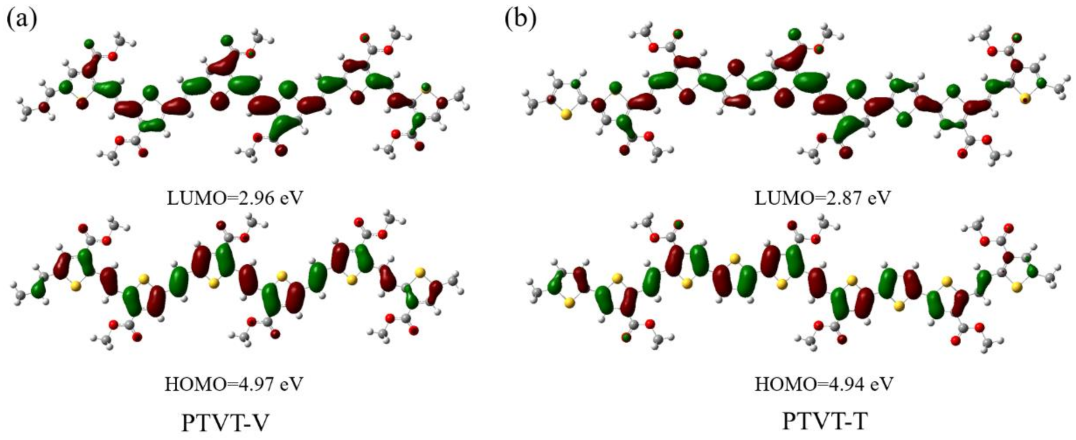

Figure S1. DFT-calculated HOMO and LUMO surfaces of the optimal geometries of (a) PTVT-V and (b) PTVT-T.

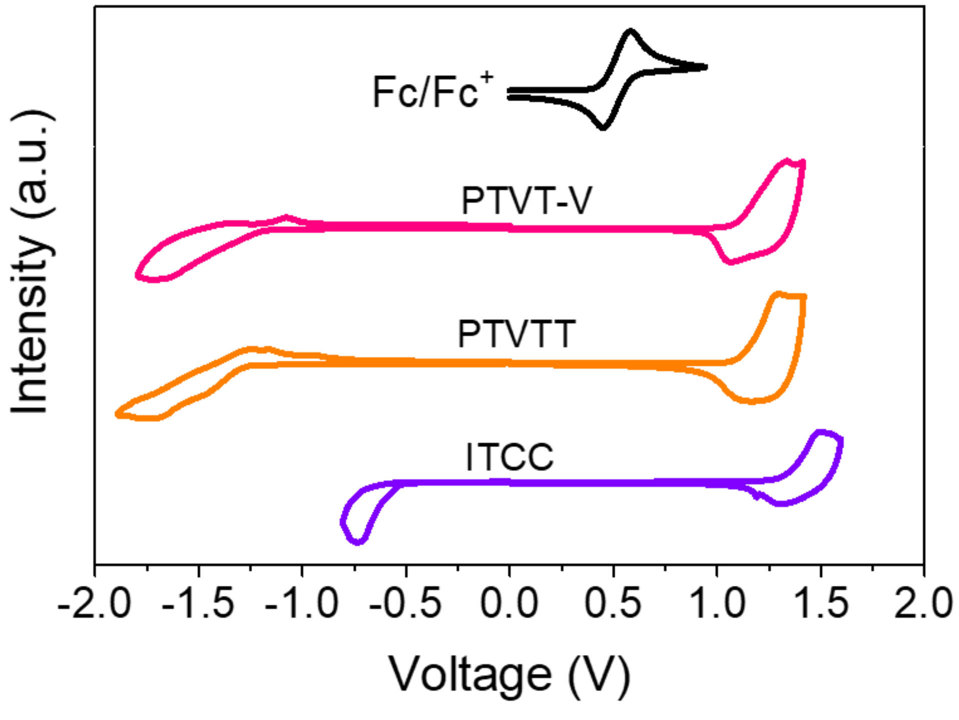

Figure S2. Cyclic voltammograms of the PTVT-V, PTVT-T, and ITCC on the Pt electrode measured with 0.1m Bu_4NPF_6 in CF solution with Ag/AgCl as the reference electrode.

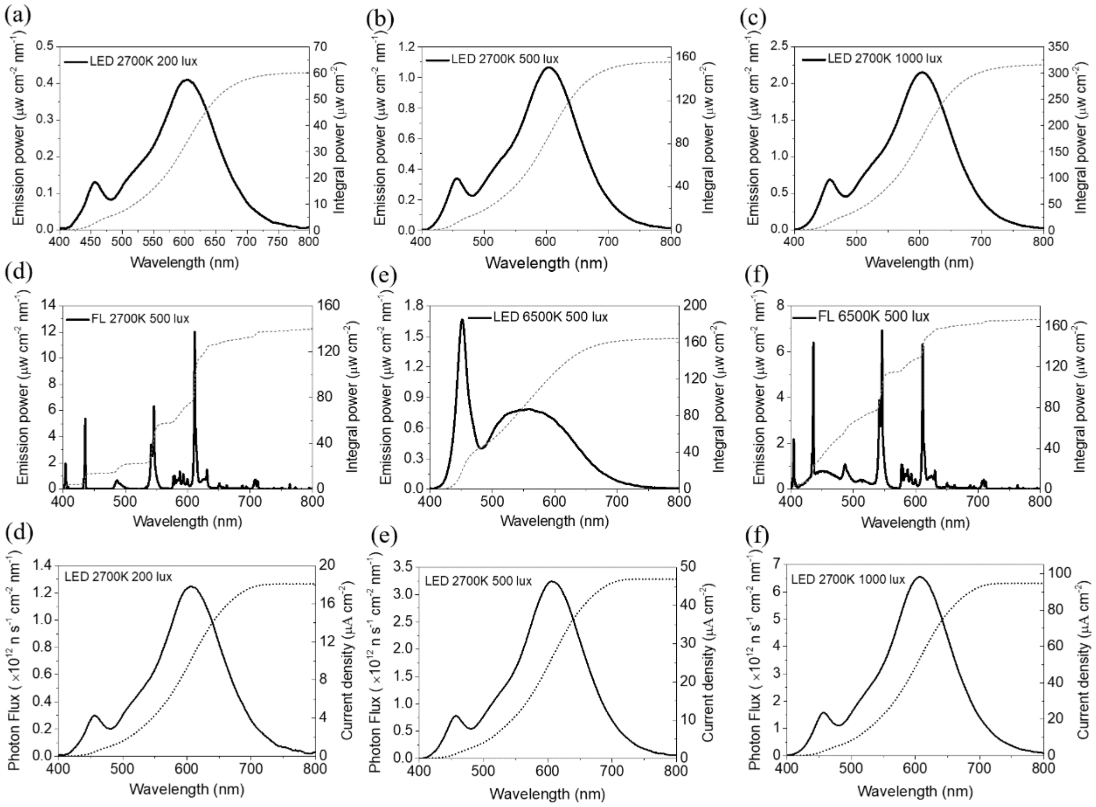

Figure S3. The emission spectra of various indoor light sources with their corresponding integrate emission powers and current density IOPV cells under LED 2700K, 200/500/1000 lux.

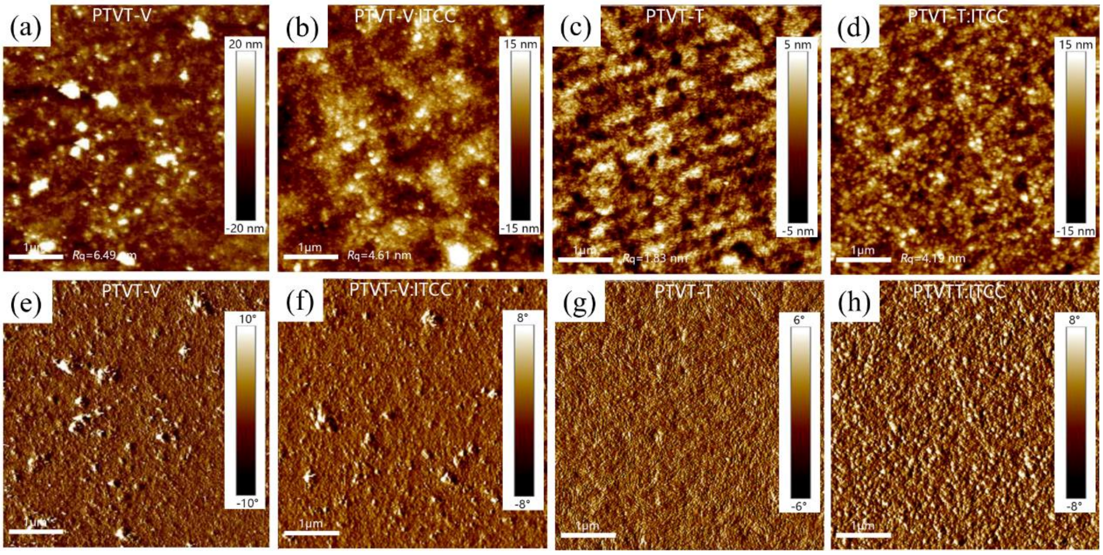

Figure S4. AFM height and phase images of both neat films PTVT-V and PTVT-T and blend films of PTVT-V:ITCC and PTVT-T:ITCC.

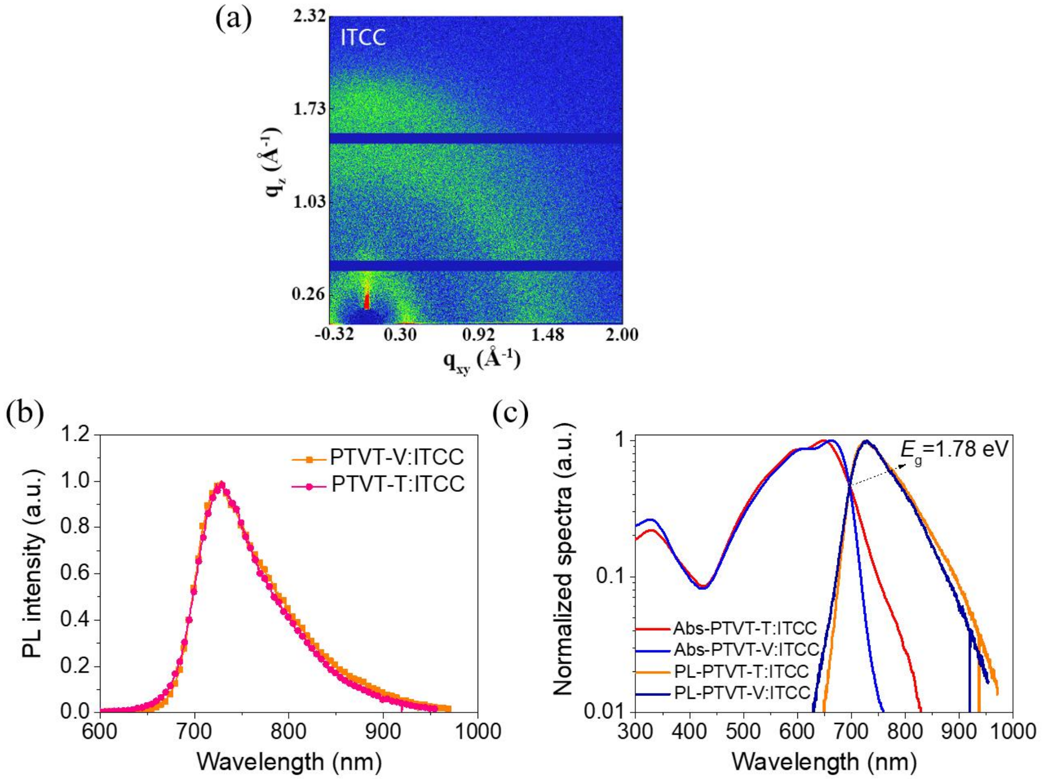

Figure S5. 2D pattern of GIWAXS of ITCC neat film. (a) Steady state PL spectra of PTVT-V:ITCC and PTVT-T:ITCC blend films. (b) The determination of E_{g}s by absorption and EL spectra of PTVT-V:ITCC and PTVT-T:ITCC blend films.

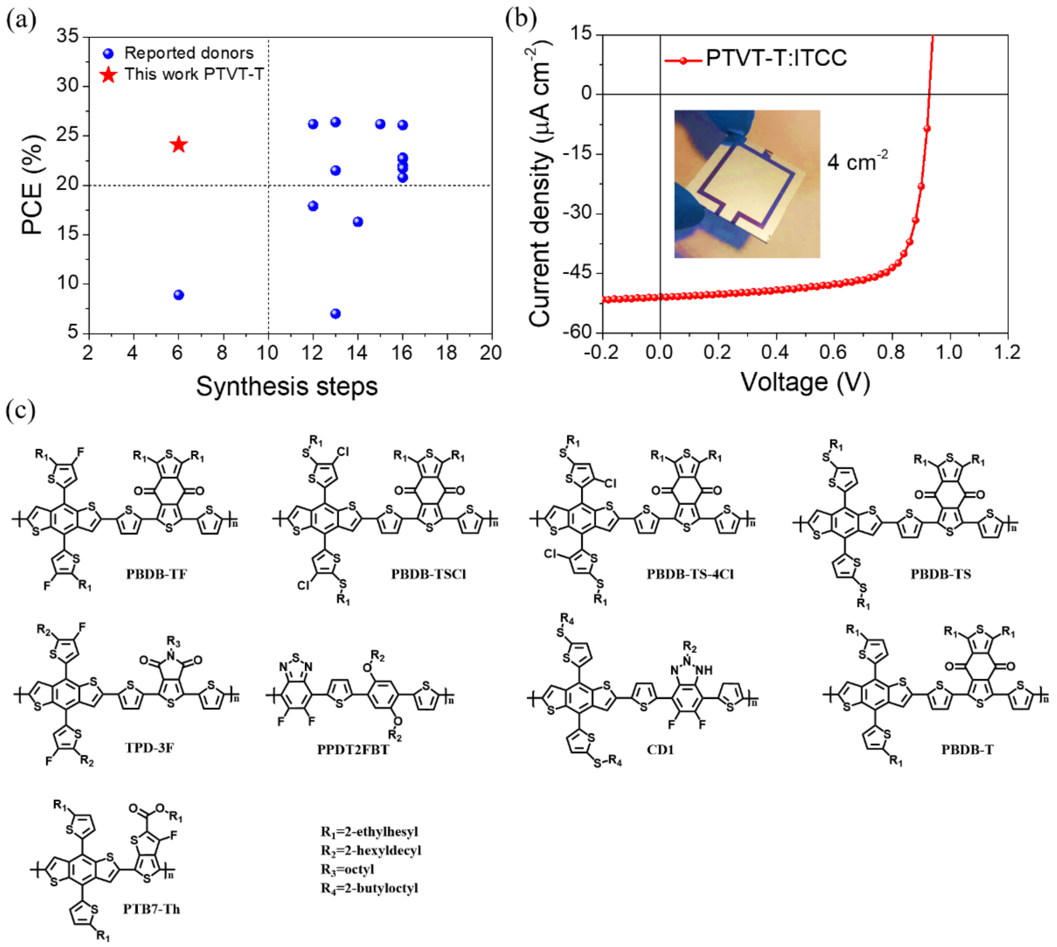

Figure S6. (a) Plots of PCE vs. synthesis steps. (b) The *J-V* curve of device based on PTVT-T:ITCC with 4 cm^{-2} area. (c) The chemical structures of the donors used in the IOPV cells mentioned in this study.

Table S1. The detailed photovoltaic parameters of PTVT-V:ITCC and PTVT-T:ITCC under various indoor light sources.

Light sources	Devices	Intensity (lux)		P_{in} ($\mu W\ cm^{-2}$)	V_{OC} (V)	J_{SC} ($\mu A\ cm^{-2}$)	FF (%)	P_{out} ($\mu W\ cm^{-2}$)	PCE[a] (%)
2700K FL	PTVT-V:ITCC	500	140	0.813	15.12	48.16	48.16	5.92	4.23 (4.18±0.04)
	PTVT-T:ITCC		140	0.926	42.71	73.81	73.81	29.19	20.85 (20.59±0.24)
6500K LED	PTVT-V:ITCC	500	160	0.813	15.93	46.56	46.56	6.03	3.77 (3.63±0.08)
	PTVT-T:ITCC		160	0.938	54.35	74.82	74.82	38.14	23.84 (23.38±0.34)
6500K FL	PTVT-V:ITCC	500	160	0.802	17.01	44.86	44.86	6.12	3.83 (3.71±0.08)
	PTVT-T:ITCC		160	0.926	50.02	73.36	73.36	33.98	21.24 (20.96±0.21)

[a)] Average values with standard deviation were obtained from 10 devices.

Table S2. Charge transport parameters of PTVT-V- and PTVT-T-based devices.

Devices	$\mu_{(0,\ 300\ K)}$ (cm^{-2} v^{-1} s^{-1})	μ_{∞} (cm^{-2} v^{-1} s^{-1})	σ (meV)
PTVT-V:ITCC	3.00×10^{-6}	7.93×10^{-5}	70
PTVT-T:ITCC	2.61×10^{-4}	2.30×10^{-3}	58

Table S3. The detailed device parameters of the IOPV cells mentioned in this study.

**Active layers**	**Donors**	**Light sources**	**Intensity (lux)**	**PCEs (%)**	**Ref.**
PBDB-TF:IO-4Cl	PBDB-TF	LED	1000	26.1	(Cui et al., 2019a)
PBDB-TF:ITCC	PBDB-TF	LED	1000	22.0	(Cui et al., 2019b)
PBDB-TF:IT-4F	PBDB-TF	LED	1000	20.8	(Cui et al., 2019b)
PBDB-TSCl:IT-4F	PBDB-TSCl	FL	500	21.5	(Park et al., 2020)
PBDB-TS-4Cl:IT-4F	PBDB-TS-4Cl	FL	500	21.7	(Je et al., 2020)
PBDB-TS:IT-4F	PBDB-TS	FL	1000	7	(Je et al., 2020)
TPD-3F:IT-4F	TPD-3F	FL	1000	26.2	(Liao et al., 2020)
PPDT2FBT:IT-M	PPDT2FBT	LED	1000	8.9	(Dayneko et al., 2019)
PBDB-TF:IT-M	PBDB-TF	LED	500	22.8	(Shrotriya et al., 2006)
CD1:PBN-10	CD1	FL	1000	26.2	(Wetzelaer et al., 2012)
CD1:ITIC	CD1	FL	1000	17.9	(Wetzelaer et al., 2012)
PBDB-T:ITICTh:$PC_{71}BM$	PBDB-T	LED	1000	26.4	(Nam et al., 2019a)

PBDB-TF:Y6:Y-Th_2	PBDB-TF	LED	1000	22.7	(Cho et al., 2020)
PTB7-Th:PBDBTF:ITICTh:PC_{71}BM	PTB7-Th	LED	1000	16.3	(Nam et al., 2019b)

References

Bi, P., Ren, J., Zhang, S., Wang, J., and Hou, J. (2020). PTV-based p-type organic semiconductors: Candidates for low-cost photovoltaic donors with simple synthetic routes. *Polymer* 209**,** 122900. doi: https://doi.org/10.1016/j.polymer.2020.122900.

Cho, Y., Kumari, T., Jeong, S., Lee, S.M., Jeong, M., Lee, B., et al. (2020). Guest-oriented non-fullerene acceptors for ternary organic solar cells with over 16.0% and 22.7% efficiencies under one-sun and indoor light. *Nano Energy* 75**,** 104896. doi: https://doi.org/10.1016/j.nanoen.2020.104896.

Cui, Y., Wang, Y., Bergqvist, J., Yao, H., Xu, Y., Gao, B., et al. (2019a). Wide-gap non-fullerene acceptor enabling high-performance organic photovoltaic cells for indoor applications. *Nat. Energy* 4(9)**,** 768-775. doi: 10.1038/s41560-019-0448-5.

Cui, Y., Yao, H., Zhang, T., Hong, L., Gao, B., Xian, K., et al. (2019b). 1 cm2 organic photovoltaic cells for indoor application with over 20% efficiency. *Adv. Mater.* 31(42)**,** 1904512. doi: https://doi.org/10.1002/adma.201904512.

Dayneko, S.V., Pahlevani, M., and Welch, G.C. (2019). Indoor photovoltaics: photoactive material selection, greener ink formulations, and slot-die coated active layers. *ACS Appl. Mater. Inter.* 11(49)**,** 46017-46025. doi: 10.1021/acsami.9b19549.

Je, H.-I., Shin, E.-Y., Lee, K.J., Ahn, H., Park, S., Im, S.H., et al. (2020). Understanding the performance of organic photovoltaics under indoor and outdoor conditions: effects of chlorination of donor polymers. *ACS Appl. Mater. Inter.* 12(20)**,** 23181-23189. doi: 10.1021/acsami.0c02712.

Liao, C.-Y., Chen, Y., Lee, C.-C., Wang, G., Teng, N.-W., Lee, C.-H., et al. (2020). Processing strategies for an organic photovoltaic module with over 10% efficiency. *Joule* 4(1)**,** 189-206. doi: https://doi.org/10.1016/j.joule.2019.11.006.

Nam, M., Kang, J.-h., Shin, J., Na, J., Park, Y., Cho, J., et al. (2019a). Ternary organic blend approaches for high photovoltaic performance in versatile applications. *Adv. Energy Mater.* 9(38)**,** 1901856. doi: https://doi.org/10.1002/aenm.201901856.

Nam, M., Noh, H.Y., Cho, J., Park, Y., Shin, S.-C., Kim, J.-A., et al. (2019b). All-day operating quaternary blend organic photovoltaics. *Adv. Funct. Mater.* 29(16)**,** 1900154. doi: https://doi.org/10.1002/adfm.201900154.

Park, S., Ahn, H., Kim, J.-y., Park, J.B., Kim, J., Im, S.H., et al. (2020). High-performance and stable nonfullerene acceptor-based organic solar cells for indoor to outdoor light. *ACS Energy Lett.* 5(1)**,** 170-179. doi: 10.1021/acsenergylett.9b01819.

Shrotriya, V., Li, G., Yao, Y., Moriarty, T., Emery, K., and Yang, Y. (2006). Accurate measurement and characterization of organic solar cells. *Adv. Funct. Mater.* 16(15)**,** 2016-2023. doi: https://doi.org/10.1002/adfm.200600489.

Wetzelaer, G.-J.A.H., Kuik, M., and Blom, P.W.M. (2012). Identifying the nature of charge recombination in organic solar cells from charge-transfer state electroluminescence. *Adv. Energy. Mater.* 2(10)**,** 1232-1237. doi: https://doi.org/10.1002/aenm.201200009.
